# Three-Component Synthesis of Some New Coumarin Derivatives as Anticancer Agents

**DOI:** 10.3389/fchem.2021.762248

**Published:** 2022-01-25

**Authors:** Latifah A. Alshabanah, Laila A. Al-Mutabagani, Sobhi M. Gomha, Hoda A. Ahmed

**Affiliations:** ^1^ Department of Chemistry, College of Science, Princess Nourah Bint Abdulrahman University, Riyadh, Saudi Arabia; ^2^ Department of Chemistry, Faculty of Science, Cairo University, Cairo, Egypt; ^3^ Chemistry Department, Faculty of Science, Islamic University of Madinah, Madinah, Saudi Arabia; ^4^ Chemistry Department, College of Sciences, Taibah University, Yanbu, Saudi Arabia

**Keywords:** acetylcoumarin, hydrazonoyl halides, multicomponent synthesis, ultrasonic radiation, anticancer

## Abstract

A three-component reaction for the synthesis of novel 3-heteroaryl-coumarin utilizing acetylcoumarin synthon under ultrasonic irradiation was developed using chitosan-grafted poly(vinylpyridine) as an eco-friendly catalyst. The process is a simple, facile, efficient procedure for the preparation of compounds displaying a thiazole ring linked to coumarin moiety. Moreover, all the products were evaluated for their anticancer activities against HEPG2-1. The results revealed that three new compounds showed promising anticancer activities.

## Introduction

Today, the second cause of death in the world is cancer (Gomes et al., 2011). Chemotherapy has become one of the important methods for cancer treatment. The identification of novel, more potent, selective, and less toxic antitumor agents is the main aim for the researchers due to its widespread, rapid development and the severe infection of the tumor diseases. In the efforts to offer suitable anticancer drugs, medicinal researchers have focused on coumarin systems.

Coumarin is a naturally occurring material as well as a versatile synthetic scaffold exhibiting a wide spectrum of biological impacts including potential anticancer activities (Vosooghi et al., 2010) such as seselin (skin cancer) (Nishino et al., 1990), acronycin (lung, colon, and ovarian cancers) (Thakur et al., 2015), calanone (leukemia and cervical carcinoma) (Emami and Dadashpour, 2015), and tephrosin (lung cancer) (Lin et al., 2014). In addition, coumarin derivatives have a tremendous ability to regulate a diverse range of cellular pathways that can be explored for their selective anticancer activities (Geisler et al., 2011; Saidu et al., 2012). Moreover, the biological evaluations of coumarins revealed that the engrossment of innumerable pathways *via* coumarins acts as anticancer agents. They target a number of pathways in cancer like as kinase inhibition, cell cycle arrest, heat shock protein (HSP90) inhibition, angiogenesis inhibition, monocarboxylate transporters inhibition, antimitotic activity, carbonic anhydrase inhibition, telomerase inhibition, aromatase inhibition, and sulfatase inhibition (Ekowati et al., 2010; Li et al., 2010; Wang et al., 2011; Bhattarai et al., 2021).

**FIGURE 1:**
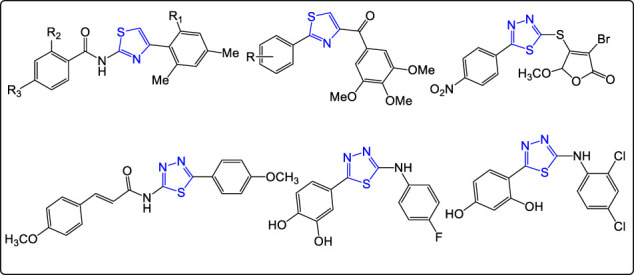
Lead compounds among thiazoles and thiadiazoles with anticancer activities.

**FIGURE 2:**
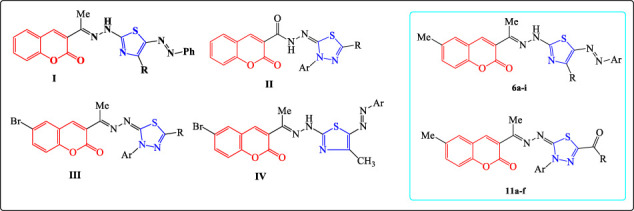
Coumarin-based thiazoles or thiadiazoles with anticancer activities and the targeted compounds.

Many investigations indicated that 1,3-thiazole derivatives possessed potential anticancer activities against various cancer types (Figure 1) (Luzina and Popov, 2009; Kashyap et al., 2018; Popsavin et al., 2007; Mavrova et al., 2009). Moreover, researchers found that the 1,3,4-thiadiazole derivatives exhibited anticancer activities with excellent IG_50_ and IC_50_ (Figure 1) (Liaras et al., 2018; Matysiak and Opolski, 2006; Kumar et al., 2010; Bhole and Bhusari, 2010). Many reports also revealed that the link between thiazole or thiadiazole groups and the coumarin moiety has broad spectra of pharmacological activities especially antitumor activities (Figure 2) (Gomha et al., 2015a; Gomha et al., 2015b; Gomha and Abdel-Aziz, 2015).

Multicomponent reactions (MCR) are one-pot mechanisms that produce a single product with at least three components, combining most or all of the starting materials (Marcaccini et al., 2003; Shaabani et al., 2008; Bachman et al., 2012; Gomha and Riyadh, 2014; Mamaghani and Hossein Nia, 2021), attributed to their reaction simplicity and high efficiency compared with multistage procedures. Furthermore, the ultrasound irradiation technique has been accomplished as an efficient heating source for organic reactions in synthetic organic chemistry. The main advantages of ultrasound-assisted reactions are shorter reaction time, simple experimental procedure, high yields, more selectivity, and clean processes (Xu et al., 2007; Jarag et al., 2011; Singh et al., 2013). One of the beneficial effects of ultrasound irradiation is playing a vital role in chemistry, especially in cases where classical tools require drastic conditions or long reactions times (Cravotto and Cintas, 2006; Cravotto et al., 2010; Pizzuti et al., 2010).

Chitosan is generated by alkaline hydrolysis of chitin (Gupta and Ravi Kumar, 2000). It is the naturally occurring copolymer polysaccharide including both glucosamine and acetylglucosamine units. Chitosan is used, in heterocyclic synthesis, as a heterogeneous phase transfer basic biocatalyst (Guibal, 2005; Qin et al., 2012; Watile and Bhanage, 2012; Alshabanah et al., 2021). Chitosan’s key drawback is that it is extremely hygroscopic and can form gels, making it difficult to recycle from the reaction mixture. To overcome this limitation, chitosan-grafted poly(vinylpyridine) has been used as a basic biocatalyst with high catalytic activities (Fu et al., 2011), which can be easily recycled and has a better basic character owing to the presence of pyridine rings.

In the light of the above findings and in continuation of our efforts to synthesize new antitumor compounds (Abbas et al., 2015; Gomha et al., 2015c; Dawood and Gomha, 2015; Gomha et al., 2016a; Gomha et al., 2016b; Gomha et al., 2017a; Gomha et al., 2017b; Tao et al., 2018; Gomha et al., 2021), the aim of the present work is to design and synthesize thiazoles and thiadiazoles linked to position 3 of coumarin as novel 3-azolylcoumarins as expected anticancer agents, utilizing sonication technique and using chitosan-grafted poly(vinylpyridine) as an eco-friendly catalyst.

## Methods and Methodology

The mass spectra were recorded on GCMS-Q1000-EX Shimadzu and GCMS 5988-A HP spectrometers, and the ionizing voltage was 70 eV (Tokyo, Japan). The IR spectra were recorded in potassium bromide discs on Shimadzu FT IR 8101 PC infrared spectrophotometer (Shimadzu, Tokyo, Japan). The ^1^H- and ^13^C-NMR spectra were recorded on Varian Mercury VXR-300 spectrometer (300 MHz for ^1^H-NMR and 75 MHz for ^13^C-NMR), and the chemical shifts were related to those of the solvent DMSO-*d*_6_ (Varian, Inc., Karlsruhe, Germany). All reactions were followed by thin-layer chromatography (TLC) (silica gel, Aluminum Sheets 60 F254, Merck, Cairo, Egypt). Elemental analyses were carried out at the Microanalytical Centre of Cairo University, Giza, Egypt. Sonication was performed in Shanghai Branson-CQX ultrasonic cleaner at a frequency of 40 kHz, and ultrasonic power was kept at 250 W.

### General Procedure for Synthesis of 1,3-Thiazole Derivatives **6a**–**i**

Method A**:** Triethylamine (TEA) (0.07 ml) was added to a mixture of the appropriate hydrazonoyl halides **5a**–**i** (1 mmol), thiosemicarbazide **4** (0.091 g, 1 mmol), and 3-acetyl-6-methyl-2*H*-chromen-2-one (**3**) (0.202 g, 1 mmol) in 20 ml of dioxane. The formed solution was irradiated by an ultrasonic generator in a water bath at 50°C for 20–60 min. Irradiation was continued till all of the starting materials have disappeared and the product was formed, monitored by TLC. The red precipitate that formed after cooling was filtered off, washed with EtOH, dried, and recrystallized from dimethylformamide (DMF) to give the corresponding thiazoles **6a**–**i**. The physical constants of products **6a**–**i** are listed below.

Method B: A mixture of equimolar amounts of **3**, **4** and the appropriate **5a**–**i** (1 mmol each) in dioxane (10 ml) containing chitosan (10 mol%) was irradiated by an ultrasonic generator in a water bath at 50°C for 20–60 min (monitored by TLC). The hot solution was filtered to remove chitosan, and excess solvent was removed under reduced pressure. The reaction mixture was triturated with methanol, and the product separated was filtered, washed with methanol, dried, and recrystallized from DMF to give compounds **6a**–**i**.

Method C: The same procedure in method B using grafted chitosan (10 mol%) instead of chitosan.

#### 6-Methyl-3-(1-(2-(4-methyl-5-(phenyldiazenyl)thiazol-2-yl)hydrazono)ethyl)-2*H*-chromen-2-one **(6a)**

Red solid, m.p. 183°C–185°C; IR (KBr) *ν* cm^−1^: 3,427 (NH), 1,724 (C═O), 1,602 (C═N); ^1^H-NMR (DMSO-*d*_6_) *δ*: 2.13 (s, 3H, CH_3_), 2.44 (s, 3H, CH_3_), 2.67 (s, 3H, CH_3_), 6.63–7.83 (m, 8H, Ar–H), 8.29 (s, 1H, coumarin-H4), 11.16 (s, br, 1H, NH) ppm; ^13^C-NMR (DMSO-*d*_6_): *δ* 11.3, 16.2, 20.1 (CH_3_), 115.6, 118.5, 125.4, 127.5, 127.8, 128.2, 128.7, 132.9, 133.1, 133.5, 133.9, 140.5, 140.8, 146.0, 151.4 (Ar–C), 168.1 (C═O) ppm; MS *m*/*z* (%): 417.13 (M^+^, 63), 351 (42), 299 (69), 247 (41), 93 (22), 80 (100), 64 (70). Anal. calcd for C_22_H_19_N_5_O_2_S (417.49): C, 63.29; H, 4.59; N, 16.78. Found: C, 63.21; H, 4.50; N, 16.69%.

#### 6-Methyl-3-(1-(2-(4-methyl-5-(*p*-tolyldiazenyl)thiazol-2-yl)hydrazono)ethyl)-2*H*-chromen-2-one **(6b)**

Red solid, m.p. 170°C–172°C; IR (KBr) *ν* cm^−1^: 3,428 (NH), 1,729 (C═O), 1,600 (C═N); ^1^H-NMR (DMSO-*d*_6_) *δ*: 2.13 (s, 3H, CH_3_), 2.38 (s, 3H, CH_3_), 2.44 (s, 3H, CH_3_), 2.73 (s, 3H, CH_3_), 6.65–7.71 (m, 7H, Ar–H), 8.29 (s, 1H, coumarin-H4), 11.18 (s, br, 1H, NH) ppm; MS *m*/*z* (%): 431 (M^+^, 100), 365 (40), 313 (26), 214 (27), 106 (53), 90 (100), 65 (70). Anal. calcd for C_23_H_21_N_5_O_2_S (431.51): C, 64.02; H, 4.91; N, 16.23. Found: C, 64.00; H, 4.83; N, 16.14%.

#### 6-Methyl-3-(1-(2-(4-methyl-5-(*m*-tolyldiazenyl)thiazol-2-yl)hydrazono)ethyl)-2*H*-chromen-2-one **(6c)**

Red solid, m.p. 183°C–185°C; IR (KBr) *ν* cm^−1^: 3,426 (NH), 1,723 (C═O), 1,609 (C═N); ^1^H-NMR (DMSO-*d*_6_) *δ*: 2.13 (s, 3H, CH_3_), 2.26 (s, 3H, CH_3_), 2.39 (s, 3H, CH_3_), 2.75 (s, 3H, CH_3_), 6.65–7.66 (m, 7H, Ar–H), 8.29 (s, 1H, coumarin-H4), 10.86 (s, br, 1H, NH) ppm; MS *m*/*z* (%): 431 (83), 384 (41), 214 (23), 106 (87), 90 (100), 65 (76). Anal. calcd for C_23_H_21_N_5_O_2_S (431.51): C, 64.02; H, 4.91; N, 16.23. Found: C, 64.01; H, 4.84; N, 16.06%.

#### 3-(1-(2-(5-((4-Methoxyphenyl)diazenyl)-4-methylthiazol-2-yl)hydrazono)ethyl)-6-methyl-2*H*-chromen-2-one **(6d)**

Red solid, m.p. 162°C–164°C; IR (KBr) *ν* cm^−1^: 3,416 (NH), 1,724 (C═O), 1,604 (C═N); ^1^H-NMR (DMSO-*d*_6_) *δ*: 2.13 (s, 3H, CH_3_), 2.38 (s, 3H, CH_3_), 2.70 (s, 3H, CH_3_), 3.84 (s, 3H, OCH_3_), 6.64–7.77 (m, 7H, Ar–H), 8.29 (s, 1H, coumarin-H4), 11.15 (s, br, 1H, NH) ppm; MS *m*/*z* (%): 447 (M^+^, 42), 431 (47), 342 (36), 241 (19), 108 (46), 80 (100), 64 (92). Anal. calcd for C_23_H_21_N_5_O_3_S (447.51): C, 61.73; H, 4.73; N, 15.65. Found: C, 61.63; H, 4.59; N, 15.60%.

#### 3-(1-(2-(5-((4-Chlorophenyl)diazenyl)-4-methylthiazol-2-yl)hydrazono)ethyl)-6-methyl-2*H*-chromen-2-one **(6e)**

Red solid, m.p. 197°C–199°C; IR (KBr) *ν* cm^−1^: 3,419 (NH), 1,726 (C═O), 1,608 (C═N); ^1^H-NMR (DMSO-*d*_6_) *δ*: 2.13 (s, 3H, CH_3_), 2.39 (s, 3H, CH_3_), 2.74 (s, 3H, CH_3_), 6.65–7.88 (m, 7H, Ar–H), 8.29 (s, 1H, coumarin-H4), 11.08 (s, br, 1H, NH) ppm; MS *m*/*z* (%): 451 (M^+^, 73), 395 (40), 214 (18), 127 (97), 80 (91), 64 (100). Anal. calcd for C_22_H_18_ClN_5_O_2_S (451.93): C, 58.47; H, 4.01; N, 15.50. Found: C, 58.36; H, 3.84; N, 15.42%.

#### 3-(1-(2-(5-((4-Bromophenyl)diazenyl)-4-methylthiazol-2-yl)hydrazono)ethyl)-6-methyl-2*H*-chromen-2-one **(6f)**

Orange solid, m.p. 170°C–173°C; IR (KBr) *ν* cm^−1^: 3,422 (NH), 1,723 (C═O), 1,601 (C═N); ^1^H-NMR (DMSO-*d*_6_) *δ*: 2.12 (s, 3H, CH_3_), 2.39 (s, 3H, CH_3_), 2.74 (s, 3H, CH_3_), 6.67–7.79 (m, 7H, Ar–H), 8.29 (s, 1H, coumarin-H4), 10.68 (s, br, 1H, NH) ppm; MS *m*/*z* (%): 496 (M^+^, 49), 377 (53), 214 (27), 171 (48), 92 (86), 65 (100). Anal. calcd for C_22_H_18_BrN_5_O_2_S (496.38): C, 53.23; H, 3.66; N, 14.11. Found: C, 53.04; H, 3.49; N, 14.02%.

#### 6-Methyl-3-(1-(2-(4-methyl-5-((4-nitrophenyl)diazenyl)thiazol-2-yl)hydrazono)ethyl)-2*H*-chromen-2-one **(6g)**

Brown solid, m.p. 213°C–215°C; IR (KBr) *ν* cm^−1^: 3,425 (NH), 1,729 (C═O), 1,603 (C═N); ^1^H-NMR (DMSO-*d*_6_) *δ*: 2.25 (s, 3H, CH_3_), 2.45 (s, 3H, CH_3_), 2.63 (s, 3H, CH_3_), 6.65–8.21 (m, 7H, Ar–H), 8.30 (s, 1H, coumarin-H4), 11.09 (s, br, 1H, NH) ppm; MS *m*/*z* (%): 462 (M^+^, 32), 375 (27), 214 (100), 138 (37), 108 (39), 65 (73). Anal. calcd for C_22_H_18_N_6_O_4_S (462.48): C, 57.14; H, 3.92; N, 18.17. Found: C, 57.04; H, 3.83; N, 18.03%.

#### 3-(1-(2-(5-((2,4-Dichlorophenyl)diazenyl)-4-methylthiazol-2-yl)hydrazono)ethyl)-6-methyl-2*H*-chromen-2-one **(6h)**

Red solid, m.p. 191°C–193°C; IR (KBr) *ν* cm^−1^: 3,426 (NH), 1,725 (C═O), 1,604 (C═N); ^1^H-NMR (DMSO-*d*_6_) *δ*: 2.13 (s, 3H, CH_3_), 2.38 (s, 3H, CH_3_), 2.73 (s, 3H, CH_3_), 6.64–7.79 (m, 6H, Ar–H), 8.29 (s, 1H, coumarin-H4), 10.73 (s, br, 1H, NH) ppm; MS *m*/*z* (%): 486 (M^+^, 73), 451 (60), 357 (48), 214 (29), 161 (85), 80 (71), 64 (100). Anal. calcd for C_22_H_17_Cl_2_N_5_O_2_S (486.37): C, 54.33; H, 3.52; N, 14.40. Found: C, 54.27; H, 3.50; N, 14.27%.

#### 6-Methyl-3-(1-(2-(5-(phenyldiazenyl)-4-(thiophen-2-yl)thiazol-2-yl)hydrazono)ethyl)-2*H*-chromen-2-one **(6i)**

Orange solid, m.p. 166°C–168°C; IR (KBr) *ν* cm^−1^: 3,420 (NH), 1,727 (C═O), 1,605 (C═N); ^1^H-NMR (DMSO-*d*_6_) *δ*: 2.18 (s, 3H, CH_3_), 2.39 (s, 3H, CH_3_), 6.68–8.06 (m, 11H, Ar–H), 8.39 (s, 1H, coumarin-H4), 11.50 (s, br, 1H, NH) ppm; MS *m*/*z* (%): 485 (M^+^, 27), 456 (83), 383 (69), 214 (18), 135 (14), 111 (100), 77 (79). Anal. calcd for C_25_H_19_N_5_O_2_S_2_ (485.58): C, 61.84; H, 3.94; N, 14.42. Found: C, 61.71; H, 3.88; N, 14.36%.

### Alternate Synthesis of **6a**

i) Synthesis of 2-(1-(6-methyl-2-oxo-2*H*-chromen-3-yl)ethylidene)hydrazine-1-carbothioamide **(7)**. A catalytic amount of concentrated hydrochloric acid was added to a mixture of 3-acetyl-6-methyl-2*H*-chromen-2-one (**3**) (2.02 g, 10 mmol) and thiosemicarbazide **4** (0.91 g, 10 mmol) in 50 ml of ethanol. The reaction mixture was irradiated by an ultrasonic generator in a water bath at 50°C for 30 min. The precipitate that formed after cooling was filtered, washed with ethanol, and recrystallized from acetic acid to give a pure product of compound **7** as yellowish-white solid in 72% yield; m.p. 231°C–233°C; IR (KBr) *ν* cm^−1^: 3,431, 4,237, 3,159 (NH and NH_2_), 1,726 (C═O), 1,604 (C═N); ^1^H-NMR (DMSO-*d*_6_) *δ*: 2.25 (s, 3H, CH_3_), 2.37 (s, 3H, CH_3_), 7.30–7.53 (m, 3H, Ar–H), 7.92 (s, 1H, coumarin-H4), 8.37 (s, br, 2H, NH_2_), 10.40 (s, br, 1H, NH) ppm; MS *m*/*z* (%): 275 (M^+^, 63), 214 (49), 175 (29), 111 (100), 77 (69), 63 (82). Anal. calcd for C_13_H_13_N_3_O_2_S (275.33): C, 56.71; H, 4.76; N, 15.26. Found: C, 56.58; H, 4.71; N, 15.09%.

#### Reaction of thiosemicarbazone 7 with 2-oxo-*N*-phenylpropanehydrazonoyl chloride **(5a)**

Chitosan-grafted poly(vinylpyridine) (0.1 g) was added to a stirred mixture of thiosemicarbazone **7** (0.275 g, 1 mmol) and 2-oxo-*N*-phenylpropanehydrazonoyl chloride (**5a**) (0.196 g, 1 mmol) in dioxane (15 ml). The reaction mixture was irradiated by an ultrasonic generator in a water bath at 40°C for 30 min. The hot solution was filtered to remove g-chitosan and excess solvent was removed under reduced pressure. The reaction mixture was triturated with methanol, and the product separated was filtered, washed with methanol, dried, and recrystallized from DMF to give the corresponding product **6a**, which was identical in all aspects (m.p., mixed m.p., and IR spectra) with those obtained from the one-pot synthesis of **3** + **4** + **5a**.

### General Procedure for Synthesis of 1,3,4-Thiadiazole Derivatives **11a**–**f**

Method A: A mixture of 3-acetyl-6-methyl-2*H*-chromen-2-one (**3**) (2.02 g, 10 mmol), methyl hydrazinecarbodithioate (**10**) (0.122 g, 1 mmol), and the appropriate hydrazonoyl halides **5a**–**f** (1 mmol) in ethanol (20 ml) containing TEA (0.07 ml) was stirred at room temperature for 4–8 h. The resulting solid was collected and recrystallized from DMF to give the corresponding 1,3,4-thiadiazoles **11a**–**f**. Products **11a**–**f** together with their physical constants are listed below.

Method B: A mixture of equimolar amounts of **3**, **10**, and the appropriate **5a**–**f** (1 mmol each) in ethanol (20 ml) containing chitosan (0.1 g) was irradiated by an ultrasonic generator in a water bath at 25°C for 10–30 min (monitored by TLC). The hot solution was filtered to remove chitosan, and excess solvent was removed under reduced pressure. The reaction mixture was triturated with methanol, and the product separated was filtered, washed with methanol, dried, and recrystallized from DMF to give compounds **11a**–**f**.

Method C: The same procedure in method B using grafted chitosan (0.1 g) instead of chitosan.

#### 3-(1-((5-Acetyl-3-phenyl-1,3,4-thiadiazol-2(3*H*)-ylidene)hydrazono)ethyl)-6-methyl-2*H*-chromen-2-one **(11a)**

Yellow solid, m.p. 214°C–216°C; IR (KBr) *ν* cm^−1^: 1,726, 1,696 (2C═O), 1,602 (C═N); ^1^H-NMR (DMSO-*d*_6_) *δ*: 2.37 (s, 3H, CH_3_), 2.44 (s, 3H, CH_3_), 2.57 (s, 3H, CH_3_), 7.34–7.72 (m, 8H, Ar–H), 8.56 (s, 1H, coumarin-H4) ppm; ^13^C-NMR (DMSO-*d*_6_): *δ* 14.7, 20.1, 29.9 (CH_3_), 107.6, 115.8, 120.4, 125.5, 127.3, 127.7, 128.7, 129.5, 130.1, 134.1, 135.3, 140.0, 146.8, 152.7 (Ar–C), 168.5, 195.0 (C═O) ppm; MS *m*/*z* (%): 418 (M^+^, 42), 338 (53), 214 (4), 177 (51), 90 (62), 64 (100). Anal. calcd for C_22_H_18_N_4_O_3_S (418.47): C, 63.14; H, 4.34; N, 13.39. Found: C, 63.05; H, 4.31; N, 13.26%.

#### 3-(1-((5-Acetyl-3-(*p*-tolyl)-1,3,4-thiadiazol-2(3*H*)-ylidene)hydrazono)ethyl)-6-methyl-2*H*-chromen-2-one **(11b)**

Yellow solid, m.p. 201°C–203°C; IR (KBr) *ν* cm^−1^: 1,724, 1,699 (2C═O), 1,606 (C═N); ^1^H-NMR (DMSO-*d*_6_) *δ*: 2.25 (s, 3H, CH_3_), 2.37 (s, 3H, CH_3_), 2.41 (s, 3H, CH_3_), 2.58 (s, 3H, CH_3_), 7.31–7.63 (m, 6H, Ar–H), 7.73 (s, 1H, Ar–H), 8.57 (s, 1H, coumarin-H4) ppm; MS *m*/*z* (%): 432 (M^+^, 68), 331 (60), 186 (44), 158 (41), 91 (100), 77 (70). Anal. calcd for C_23_H_20_N_4_O_3_S (432.50): C, 63.87; H, 4.66; N, 12.95. Found: C, 63.71; H, 4.62; N, 12.84%.

#### Ethyl 5-((1-(6-methyl-2-oxo-2*H*-chromen-3-yl)ethylidene)hydrazono)-4-phenyl-4,5-dihydro-1,3,4-thiadiazole-2-carboxylate **(11c)**

Yellow solid, m.p. 190°C–192°C; IR (KBr) *ν* cm^−1^: 1,740, 1,722 (2C═O), 1,603 (C═N); ^1^H-NMR (DMSO-*d*_6_) *δ*: 1.30 (t, 3H, *J* = 6.9 Hz, CH_2_CH_3_), 2.37 (s, 3H, CH_3_), 2.62 (s, 3H, CH_3_), 4.27 (q, 2H, *J* = 6.9 Hz, CH_2_CH_3_), 7.22–7.69 (m, 7H, Ar–H), 7.73 (s, 1H, Ar–H), 8.55 (s, 1H, coumarin-H4) ppm; MS *m*/*z* (%): 448 (M^+^, 26), 338 (39), 214 (63), 158 (50), 90 (86), 77 (100). Anal. calcd for C_23_H_20_N_4_O_4_S (448.50): C, 61.60; H, 4.50; N, 12.49. Found: C, 61.52; H, 4.38; N, 12.36%.

#### Ethyl 5-((1-(6-methyl-2-oxo-2*H*-chromen-3-yl)ethylidene)hydrazono)-4-(*p*-tolyl)-4,5-dihydro-1,3,4-thiadiazole-2-carboxylate **(11d)**

Yellow solid, m.p. 171°C–173°C; IR (KBr) *ν* cm^−1^: 1,742, 1,722 (2C═O), 1,606 (C═N); ^1^H-NMR (DMSO-*d*_6_) *δ*: 1.27 (t, 3H, *J* = 6.9 Hz, CH_2_CH_3_), 2.23 (s, 3H, CH_3_), 2.37 (s, 3H, CH_3_), 2.65 (s, 3H, CH_3_), 4.29 (q, 2H, *J* = 6.9 Hz, CH_2_CH_3_), 7.33–7.56 (m, 6H, Ar–H), 7.71 (s, 1H, Ar–H), 8.55 (s, 1H, coumarin-H4) ppm; MS *m*/*z* (%): 462 (M^+^, 70), 384 (94), 331 (40), 186 (36), 91 (100), 77 (69). Anal. calcd for C_24_H_22_N_4_O_4_S (462.52): C, 62.32; H, 4.79; N, 12.11. Found: C, 62.19; H, 4.64; N, 12.03%.

#### 5-((1-(6-Methyl-2-oxo-2*H*-chromen-3-yl)ethylidene)hydrazono)-*N*,4-diphenyl-4,5-dihydro-1,3,4-thiadiazole-2-carboxamide **(11e)**

Yellow solid, m.p. 190°C–192°C; IR (KBr) *ν* cm^−1^: 3,427 (NH), 1,740, 1,679 (2C═O), 1,600 (C═N); ^1^H-NMR (DMSO-*d*_6_) *δ*: 2.37 (s, 3H, CH_3_), 2.57 (s, 3H, CH_3_), 7.34–7.57 (m, 12H, Ar–H), 7.72 (s, 1H, Ar–H), 8.56 (s, 1H, coumarin-H4), 10.63 (s, br, 1H, NH) ppm; MS *m*/*z* (%): 495 (M^+^, 49), 418 (37), 331 (41), 186 (54), 91 (100), 77 (86), 64 (61). Anal. calcd for C_27_H_21_N_5_O_3_S (495.56): C, 65.44; H, 4.27; N, 14.13. Found: C, 65.28; H, 4.21; N, 14.05%.

#### 6-Methyl-3-(1-((3-phenyl-5-(thiophene-2-carbonyl)-1,3,4-thiadiazol-2(3*H*)-ylidene)hydrazono)ethyl)-2*H*-chromen-2-one **(11f)**

Yellow solid, m.p. 240°C–242°C; IR (KBr) *ν* cm^−1^: 1,726, 1,693 (2C═O), 1,607 (C═N); ^1^H-NMR (DMSO-*d*_6_) *δ*: 2.37 (s, 3H, CH_3_), 2.64 (s, 3H, CH_3_), 7.34–7.69 (m, 10H, Ar–H), 7.71 (s, 1H, Ar–H), 8.55 (s, 1H, coumarin-H4) ppm; MS *m*/*z* (%): 486 (M^+^, 27), 426 (60), 330 (39), 111 (51), 80 (100), 64 (89). Anal. calcd for C_25_H_18_N_4_O_3_S_2_ (486.56): C, 61.71; H, 3.73; N, 11.52. Found: C, 61.58; H, 3.64; N, 11.41%.

### Alternate Synthesis of **11a**

i) Synthesis of methyl 2-(1-(6-methyl-2-oxo-2*H*-chromen-3-yl)ethylidene)hydrazine-1-carbodithioate **(12)**. To a solution of 3-acetyl-6-methyl-2*H*-chromen-2-one (**3**) (2.02 g, 10 mmol) in 2-propanol (20 ml), methyl hydrazinecarbodithioate (**10**) (1.22 g, 10 mmol) was added. The reaction mixture was irradiated by an ultrasonic generator in a water bath at 30°C for 30 min. The precipitate that formed after cooling was filtered, washed with ethanol, and recrystallized from acetic acid to give pure product of compound **12** as yellowish-white solid in 68% yield; m.p. 175°C–177°C; IR (KBr) *ν* cm^−1^: 3,420 (NH), 1,723 (C═O), 1,604 (C═N); ^1^H-NMR (DMSO-*d*_6_) *δ*: 2.25 (s, 3H, CH_3_), 2.37 (s, 3H, CH_3_), 2.57 (s, 3H, SCH_3_), 7.33 (d, *J* = 9 Hz, 1H, Ar–H), 7.54 (d, *J* = 9 Hz, 1H, Ar–H), 7.71 (s, 1H, Ar–H), 8.55 (s, 1H, coumarin-H4), 11.79 (s, br, 1H, NH) ppm; MS *m*/*z* (%): 306 (M^+^, 18), 186 (40), 158 (37), 128 (39), 90 (100), 77 (86), 64 (75). Anal. calcd for C_14_H_14_N_2_O_2_S_2_ (306.40): C, 54.88; H, 4.61; N, 9.14. Found: C, 54.70; H, 4.58; N, 9.06%.
ii) Reaction of carbodithioate 12 with 2-oxo-*N*-phenylpropanehydrazonoyl chloride **(5a)**. TEA (0.5 ml) was added to a stirred ethanolic solution of methyl hydrazinecarbodithioate (**10**) (0.122 g, 1 mmol) and 2-oxo-*N*-phenylpropanehydrazonoyl chloride (**5a**) (0.196 g, 1 mmol). The reaction mixture was irradiated by an ultrasonic generator in a water bath at 40°C for 30 min. The solid precipitated after cooling and was filtered off, washed with water, dried, and finally recrystallized from DMF to give the corresponding product **11a**, which was identical in all aspects (m.p., mixed m.p., and IR spectra) with those obtained from the one-pot synthesis of **3** + **10** + **5a.**

### Anticancer Activities

The cytotoxic evaluation of the synthesized compounds was carried out at the Regional Center for Mycology and Biotechnology at Al-Azhar University, Cairo, Egypt, according to the reported method (Gomha et al., 2015c; Gomha et al., 2015d). For more details, see the supporting information file.

## Results and Discussion

**SCHEME 1:**
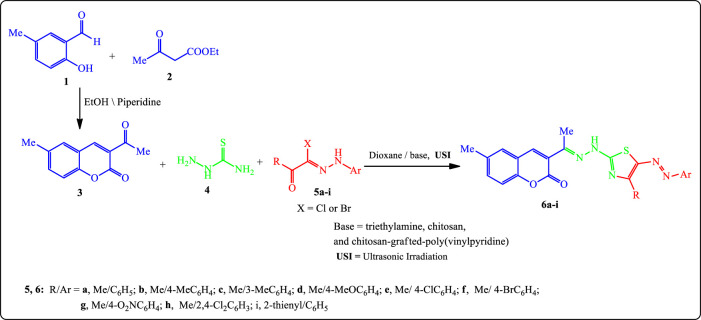
Synthesis of arylazothiazole derivatives **6a–i**.

In continuation of our previous work to synthesize bioactive heterocyclic compounds under mild conditions, herein we wish to report a mild and efficient procedure for the synthesis of some thiazolyl-coumarins *via* the one-pot, three-component reaction of 3-acetyl-6-methyl-2*H*-chromen-2-one (**3**) (prepared previously from the reaction of **1** with **2**) (Scheme 1) (Toan et al., 2020), thiosemicarbazone (**4)**, and the appropriate hydrazonoyl halides **5a**–**i** [52**]** in dioxane under ultrasonic irradiation (USI) at 50°C for 20–60 min, in the presence of different basic catalysts such as TEA, chitosan, and chitosan-grafted poly(vinylpyridine) (Scheme 1). The development of all reactions was tracked by TLC. At the outset, the identification of the best basic catalyst was examined (Table 1).

**TABLE 1:** Comparison of synthesis of thiazolylcoumarines **6a–i** under ultrasound irradiation using different basic catalysts on the time of reaction and the yield% of the products.

Compound no	TEA		Chitosan		g-Chitosan^a^	
	Time (min)	(%) Yield	Time (min)	(%) Yield	Time (min)	(%) Yield
**6a**	50	72	41	79	20	93
**6b**	50	74	43	83	23	89
**6c**	56	73	46	84	25	91
**6d**	53	72	43	84	28	94
**6e**	52	72	36	86	19	89
**6f**	60	72	38	83	25	88
**6g**	55	73	38	84	22	89
**6h**	50	72	37	84	26	90
**6i**	45	72	37	86	28	92

a g-chitosan, chitosan-grafted poly(vinylpyridine); TEA, triethylamine.

As shown from Table 1, chitosan-grafted poly(vinylpyridine) was the best choice of a basic catalyst under USI. The reaction proceeds smoothly with an electron-rich as well as electron-deficient substituent on the aromatic benzene ring of hydrazonoyl halides **5**. The structures of isolated products **6a**–**i** were evidenced by spectral data together with elemental analyses. We have observed that under the same reaction conditions, the yields of the desired products **6a**–**i** increase by changing TEA into chitosan. Moreover, using grafted chitosan as a basic catalyst has a significant increasing effect on the product yields. In addition, the heating under USI was more efficient than conventional heating, as it reduced the reaction time and increased the product yields in the case of compounds **6a**–**c** as shown in Supplementary Table S1.

The elemental analyses and spectroscopic data of the obtained products **6a**–**i** supported the assigned structures. The IR spectrum of **6a** as a representative example exhibits two strong stretching frequencies in the regions of 3,427 and 1,724 cm^−1^, attributable to the NH and C═O groups, respectively. Its ^1^H-NMR spectrum displayed five singlet signals for the 3CH_3_, coumarin-H4, and NH protons at *δ* 2.13, 2.44, 2.67, 8.29, and 11.16 ppm, in addition to the characteristic multiplet signal for the eight aromatic protons. Moreover, its ^13^C-NMR showed three aliphatic signals for the three methyl groups at 11.3, 16.2, and 20.1 ppm; 15 aromatic signals at 115.6–151.4 ppm; and one carbonyl signal at 168.1 ppm. IR (KBr) spectra showed three bands at *v* 3,422, 1,671, and 1,653 cm^−1^ assignable to the NH and 2C═O groups. The mass spectrum is also an additional evidence for supporting the obtained structure, which gave a molecular ion at *m*/*z* 417.13 [M^+^] (Gomha et al., 2015b; Gomha et al., 2015c; Alshabanah et al., 2021; Gomha et al., 2021).

**SCHEME 2:**
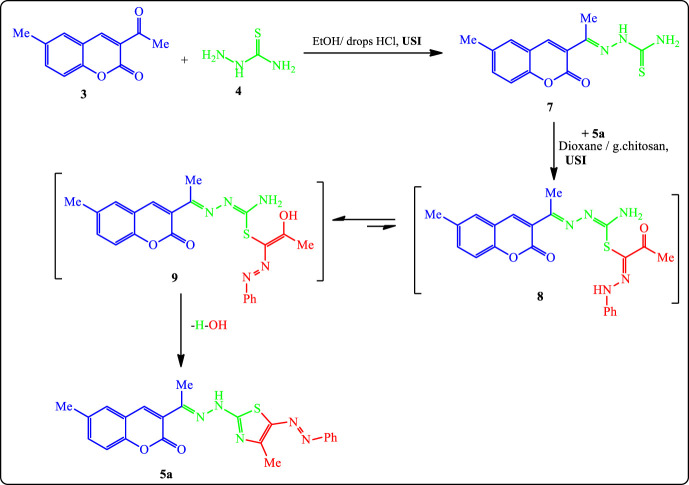
Alternative synthesis of phenyazothiazole derivative **5a**.

On the other hand, reaction of 2-(1-(6-methyl-2-oxo-2*H*-chromen-3-yl)ethylidene)hydrazine-1-carbothioamide (**7**) (prepared separately through condensation of 3-acetyl-6-methyl-2*H*-chromen-2-one (**3**) with thiosemicarbazide in ethanol containing drops of concentrated HCl) with 2-oxo-*N*-phenylpropanehydrazonoyl chloride (**5a**) (Shawali and Gomha, 2000) gave an identical product in IR, m.p., and mixed m.p. with **6a** (Scheme 2). The overall yield for this 2-step reaction is 83%.

To achieve the best experimental conditions and the different factors (such as catalyst loading, temperature, solvent, and reaction time) on the reaction of **3** + **4** + **5a** in the presence of a catalytic amount of chitosan-grafted poly(vinylpyridine) in one-pot synthesis under USI to get 6-methyl-3-(1-(2-(4-methyl-5-(phenyldiazenyl)thiazol-2-yl)hydrazono)ethyl)-2*H*-chromen-2-one (**6a**), the following was carried out.

In the first step, we examined the effect of the amount of catalyst for the synthesis of compound **6a** (Table 2, entries 1–3). The best results were obtained using 10 mol% of catalyst (93%) (Table 2, entry 3). Using lower amounts of catalyst resulted in lower yields (Table 2).

**TABLE 2:** Optimization of the reaction conditions (catalyst loading, solvent, reaction time, and temperature) for the synthesis of compound **6a**.

Entry	Catalyst (mol%)	Solvent	Time (min)	Temperature (°C)	Yield (%)
1	1	Dioxane	25	50	57
2	5	Dioxane	25	50	79
3^a^	10	Dioxane	25	50	93
4	10	EtOH	25	50	90
5	10	DMSO	25	50	88
6	10	Dioxane	20	50	89
7	10	Dioxane	30	50	93
8	10	Dioxane	25	25	80
9	10	Dioxane	25	40	88
10	10	Dioxane	25	60	93

a The best reaction condition for the synthesis of compound **6a**.

In the next step, the efficiency of the different solvents was examined under USI (Table 2, entries 3–5). Screening of various solvents showed that the formation of product **6a** proceeded in the highest yield with a higher reaction rate in dioxane (Table 2, entry 3).

Furthermore, the reaction time was examined under USI (Table 2, entries 3, 6, and 7). The best time for the formation of product **6a** was 25 min (Table 2, entry 3).

In continuation, the effect of temperatures was also tested on the reaction, and the results are presented in Table 2 (entries 3, 8, 9, and 10). According to Table 2, increasing the reaction temperature from 25°C to 40°C–60°C under USI increases the yields of products from 80% to 88%–93%, respectively. Finally, 50°C was selected as the optimum temperature (Table 2, entry 3).

As shown in Table 2, we observed that the optimum reaction conditions for the formation of product **6a** are as follows: reaction of **3** + **4** + **5a** in dioxane under USI in the presence of 10 mol% of chitosan-grafted poly(vinylpyridine) at 50°C for 25 min.

Thus, irradiation of **3** + **4** + **5b**–**i** under the optimum conditions led to the formation of 6-methyl-3-(1-(2-(4-substituted-5-(aryldiazenyl)thiazol-2-yl)hydrazono)ethyl)-2*H*-chromen-2-one derivatives **6b**–**i** (Scheme 1).

**SCHEME 3:**
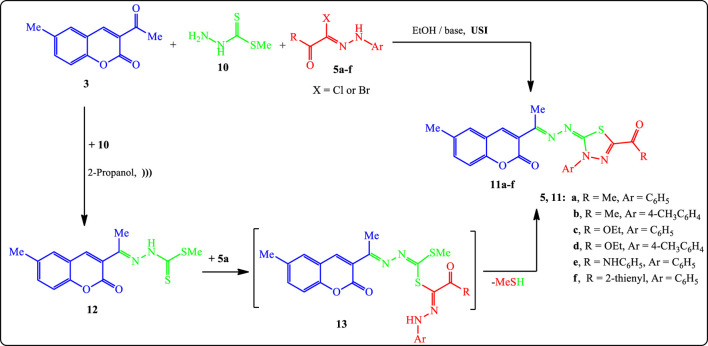
Synthesis of thiadiazole derivatives **11a–f**.

Our research has now been expanded to include the synthesis of new 1,3,4-thiadiazole derivatives in an attempt to functionalize the target 3-thiadiazolyl coumarins **11a**–**f**. Thus, treatment of compound **3**, methyl hydrazinecarbodithioate **(10)**, and various derivatives of hydrazonoyl halides **5a**–**f** in EtOH under USI at 25°C for 10–30 min in the presence of TEA or the chitosan or chitosan-grafted poly(vinylpyridine) as a basic catalyst afforded the respective 1,3,4-thiadiazoles **11a**–**f** as depicted in Scheme 3.

Table 3 shows the yield % of the isolated products **11a–f**, the g-chitosan as a basic catalyst prevailed over chitosan and TEA under sonication technique.

**TABLE 3:** Comparison of synthesis of thiadiazolylcoumarines **11a–f** under USI using different basic catalysts on the time of reaction and the yield% of the products.

Compound no.	TEA		Chitosan		g-Chitosan	
	Time (min)	(%) Yield	Time (min)	(%) Yield	Time (min)	(%) Yield
**11a**	50	72	41	79	20	91
**11b**	50	74	43	83	23	89
**11c**	56	73	46	84	25	94
**11d**	53	72	43	84	28	88
**11e**	52	72	36	86	19	90
**11f**	60	72	38	83	25	89

Note. USI, ultrasonic irradiation; TEA, trimethylamine; g-chitosan, chitosan-grafted poly(vinylpyridine).

The structures of products **11a**–**f** were elucidated based on spectral and analytical data as illustrated in the *Methods and Methodology*. For example, the IR spectra of the isolated products **11** revealed the existence of the characteristic bands for the two C═O groups at the normal wave numbers. The ^1^H-NMR spectra of compound **11a** showed the expected signals at *δ*: 2.37, 2.44, 2.57 (3s, 3CH_3_), 8.56 (s, coumarin-H4) in addition to one multiplet signal at *δ* 7.21–7.44 ppm due to eight aromatic protons. ^13^C-NMR (DMSO-*d*_6_) for compound **11a** showed the characteristic signals for 3CH_3_ and the two C═O groups at *δ* 14.7, 20.1, 29.9, 168.5, and 195.0 ppm, in addition to the expected aromatic carbons. The mass spectra of products **11a**–**f** revealed a molecular ion peak for each one, which is consistent with the respective molecular weight.

Alternatively, compound **11a** was synthesized from a reaction of 2-oxo-*N*-phenylpropanehydrazonoyl chloride (**5a**) in EtOH containing a catalytic amount of g-chitosan under USI with carbothioamide **12** (prepared separately through condensation of compound **3** with methyl hydrazinecarbodithioate **(10)** in 2-propanol). The obtained product was found to be identical to **11a** in all regards (m.p., TLC, and IR spectrum), which provides additional evidence to all **11a**–**f** structures. The overall yield for this 2-step process was 78%.

Also, one-pot synthesis of **3** + **10** + **5a** under USI in the presence of a catalytic amount of chitosan-grafted poly(vinylpyridine) was examined at different temperatures, solvents, and reaction times. This reaction led to the formation of 1,3,4-thiadiazole **11a**.

In the first step, we examined the effect of different solvents under USI (Table 4, entries 1–3). Screening of various solvents showed that the formation of product **11a** proceeded in the highest yield with a higher reaction rate in ethanol (Table 4, entry 1).

**TABLE 4:** Optimization of the reaction conditions (solvent, reaction time, and temperature) for the synthesis of compound **11a**.

Entry	Catalyst (mol%)	Solvent	Time (min)	Temperature (°C)	Yield (%)
1^a^	10	EtOH	15	25	91
2	10	Dioxane	15	25	81
3	10	DMSO	15	25	83
4	10	EtOH	10	25	86
5	10	EtOH	20	25	91
6	10	EtOH	15	50	91

a The best reaction condition for the synthesis of compound **11a**.

We also examined the reaction time under USI (Table 4, entries 1, 4, and 5). The best time for the formation of product **11a** is 15 min (Table 4, entry 1).

Finally, we also tested the reaction temperatures, and the results are presented in Table 4 (entries 1 and 6). According to Table 4, increasing the reaction temperature from 25°C to 50°C did not affect the yield of the product. Finally, 25°C was selected as the optimum temperature (Table 4, entry 1).

As shown in Table 4, we can observe that the optimum reaction conditions for the formation of product **11a** are as follows: reaction of **3** + **10** + **5a** in ethanol under USI in the presence of 0.1 g of chitosan-grafted poly(vinylpyridine) at 25°C for 15 min.

Thus, irradiation of **3** + **10** + **5b**–**i** under the optimum conditions led to formation of the respective 1,3,4-thiadiazole derivatives **11b**–**i** (Scheme 3).

Generally, the formation of products **6a**–**i** and **11a**–**f** with the application of the USI/catalytic system in a three-component one-pot reaction gave high reaction yield in short reaction duration, and the achieved results showed the tremendous synergistic effect between g-chitosan and USI.

### Antitumor Activity

**FIGURE 3:**
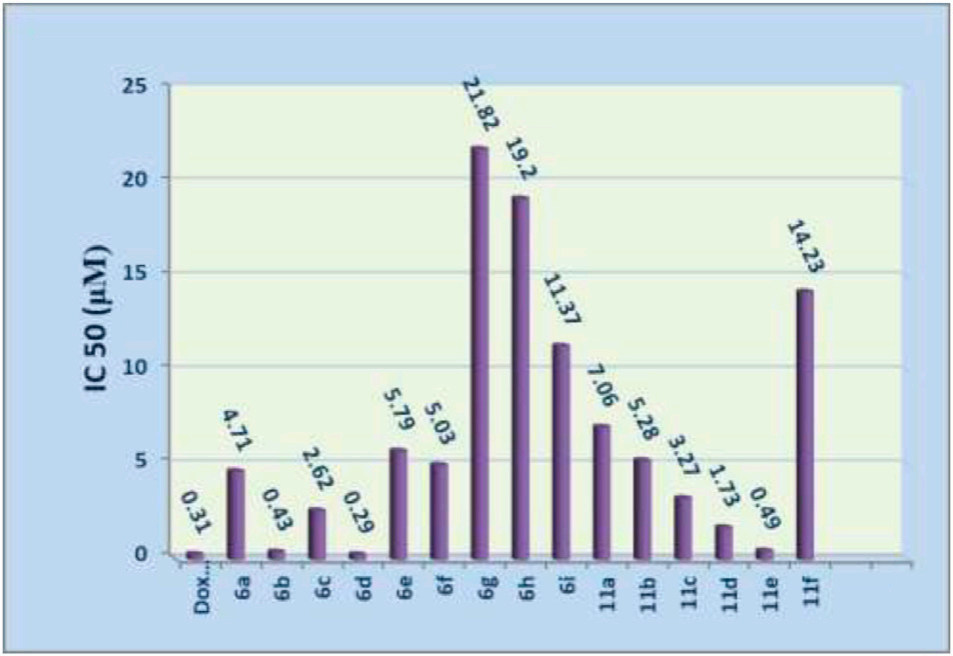
Cytotoxic activities of tested compounds against HEPG2-1.

The cytotoxic activity of the newly prepared compounds was determined against liver carcinoma cell line (HEPG2-1) using the 3-(4,5-dimethylthiazol-2-yl)-2,5-diphenyltetrazolium bromide (MTT) assay. Doxorubicin was used as a reference drug. Data generated were used to plot a dose–response curve of which the concentration (µM) of the test compounds required to kill 50% of the cell population (IC_50_) was determined. The cytotoxic activity was expressed as the mean IC_50_ of three independent experiments (Table 5 and Figure 3). The results revealed that the descending order of activity of the tested compounds was as follows: **6d** > **6b** > **11e** > **11d** > **6c** > **11c** > **6a** > **6f** > **11b** > **6e** > **11a** > **6i** > **11f** > **6h** > **6g**.

**TABLE 5:** Cytotoxic activities of tested compounds against HEPG2-1.

Compd No	R	Ar	IC_50_ (µM)
**Doxorubicin**	−	−	0.31 ± 0.48
**6a**	Me	C_6_H_5_	4.71 ± 0.72
**6b**	Me	4-MeC_6_H_4_	0.43 ± 0.66
**6c**	Me	3-MeC_6_H_4_	2.62 ± 0.75
**6d**	Me	4-MeOC_6_H_4_	0.29 ± 0.45
**6e**	Me	4-ClC_6_H_4_	5.79 ± 0.81
**6f**	Me	4-BrC_6_H_4_	5.03 ± 0.56
**6g**	Me	4-NO_2_C_6_H_4_	21.82 ± 0.79
**6h**	Me	2,4-Cl_2_C_6_H_3_	19.20 ± 0.91
**6i**	2-Thienyl	C_6_H_5_	11.37 ± 0.49
**11a**	Me	C_6_H_5_	7.06 ± 0.77
**11b**	Me	4-MeC_6_H_4_	5.28 ± 0.73
**11c**	OEt	C_6_H_5_	3.27 ± 0.48
**11d**	OEt	4-MeC_6_H_4_	1.73 ± 0.61
**11e**	NHC_6_H_5_	C_6_H_5_	0.49 ± 0.38
**11f**	2-Thienyl	C_6_H_5_	14.23 ± 0.59

### Structure–Activity Relationship

The activities of the synthesized compounds depend on the structural skeleton and electronic environment of the molecules.

Based on our limited study, the 1,3-thiazole derivatives have an *in vitro* inhibitory activity greater than that of the 1,3,4-thiadiazole derivatives: (**6a** > **11a**, **6b** > **11b** and **6i** > **11f**).

#### For the 1,3-thiazole ring **6a**–i

The *in vitro* inhibitory activity of 4-methyl-thiazole derivative **6a** was greater than that of 4-(2-thienyl)-thiazole derivative **6i**.

The introduction of an electron-donating group (methyl or methoxy groups) enhanced the antitumor activity. In contrast, the introduction of the electron-withdrawing group (chlorine or bromine or nitro group) at C4 of the phenyl group at position 4 in the 1,3-thiazole ring decreased the activity: (**6d**, **6b** > **6f**, **6e**, **6g**).

The *in vitro* inhibitory activity of the 4-tolyl-thiazole derivative **6b** was greater than that of 3-tolyl-thiazole derivative **6c**.

The *in vitro* inhibitory activity of the 4-chlorophenyl-thiazole derivative **6e** was greater than that of 2,4-dichlorophenyl-thiazole derivative **6h**.

#### For 1,3,4-thiadiazoles **11a**–**f**

The *in vitro* inhibitory activity of 5-substituted thiadiazoles was in the order of CONHPh > COOEt > CH_3_CO > 2-thienyl: (**11e** > **11c** > **11a** > **11f**).

The *in vitro* inhibitory activity of 4-methyl-thiadiazole derivative **11a** was greater than that of 4-(2-thienyl)-thiadiazole derivative **11f**.

Generally, on fixing the substituents at position 5, the electron-donating group (methyl) at C4 of the phenyl ring enhances the antitumor activity, while the electron-withdrawing group (chlorine) decreases the activity: (**11b** > **11a** and **11d** > **11c**).

## Conclusion

A new, efficient, and regioselective method for the preparation of novel 3-azolyl-coumarins by reaction of 3-acetyl-6-methyl-2*H*-chromen-2-one, thiosemicarbazide, or methyl hydrazinecarbodithioate and the appropriate hydrazonoyl halides under ultrasound irradiation at ambient temperature in a short time and high yields was developed and discussed. The assigned structure for all the newly synthesized compounds was elucidated by elemental and spectral analysis data. Moreover, the new compounds were tested *in vitro* against the HEPG2-1 cell line using the MTT viability assay. Compounds **6b**, **6d**, and **11e** have promising activities (IC_50_ value of 0.43 ± 0.66, 0.29 ± 0.45, and 0.49 ± 0.38 µM, respectively), compared with doxorubicin standard drug (IC_50_ value of 0.31 ± 0.48 µM).

## Data Availability

The original contributions presented in the study are included in the article/Supplementary Material. Further inquiries can be directed to the corresponding author.

## Abbreviations

DMF, dimethylformamide; DMSO-*d*_6_, deuterated dimethylsulfoxide; EtOH, ethanol; g-chitosan, chitosan-grafted poly(vinylpyridine); HEPG2-1, liver carcinoma cell line; MCR, multicomponent reactions; MTT, mean transit time (brain tissue blood flow); TEA, triethylamine; TLC, thin-layer chromatography; USI, ultrasonic irradiation.
