# Supplementary material for: Three-Component Synthesis of Some New Coumarin Derivatives as Anticancer Agents
Source: Front Chem. 2022 Jan 25;9:762248. doi: 10.3389/fchem.2021.762248 (PMC8822056; doi:10.3389/fchem.2021.762248)
Supplement: Supplementary file 1 [file DataSheet1.PDF]

Three-Component Synthesis of Some New Coumarin derivatives as Anti-Cancer Agents

Latifah A. Alshabanah[1], Laila A. Al-Mutabagani[1], Sobhi M. Gomha[2,3*] and Hoda A. Ahmed[2,4]

[1.] *Department of Chemistry, College of Science, Princess Nourah bint Abdulrahman University, Riyadh, Saudi Arabia.*

[2.] *Department of Chemistry, Faculty of Science, Cairo University, Cairo 12613, Egypt, ahoda@sci.cu.edu.eg, smgomha@iu.edu.sa*

[3.] *Chemistry Department, Faculty of Science, Islamic University of Madinah, Al-Madinah Al-Munawwarah 42351, Saudi Arabia.*

[4.] *Chemistry Department, College of Sciences, Yanbu, Taibah University, Yanbu 30799, Saudi Arabia.*

* *Correspondence: S.M.Gomha, s.m.gomha@gmail.com, smgomha@iu.edu.sa*

Synthesis of 1,3-thiazole derivatives 6a-c under Conventional heating

A mixture of equimolar amounts of **3, 4** and the appropriate **5a-c** (1 mmol each) in dioxane (10 mL) containing g.chitosan (10 mol%) was refluxed for 2-5 hr (monitored by TLC). The excess solvent was evaporated under reduced pressure. The reaction mixture was triturated with methanol and the product separated was filtered, washed with methanol, dried and recrystallized from DMF to give compounds **6a-c**.

Table S1. Comparison between conventional heating and USI for synthesis of thiazolylcoumarines **6a-c** using g.chitosan

Compound no.	Conventional heating		USI	
	Time (hr)	(%) yield	Time (min)	(%) yield
**6a**	3	84	20	93
**6b**	5	80	23	89
**6c**	3	82	25	91

Evaluation of Cytotoxic Effects of certain Chemical compound

Mammalian cell lines: HepG-2 cell lines were obtained from VACSERA Tissue Culture Unit.

Chemicals Used: Dimethyl sulfoxide (DMSO), crystal violet and trypan blue dye were purchased from Sigma (St. Louis, Mo., USA).

Fetal Bovine serum, DMEM, RPMI-1640, HEPES buffer solution, L-glutamine, gentamycin and 0.25% Trypsin-EDTA were purchased from Lonza (Muenchensteinerstrasse 38, CH-4002 Basel, Switzerland).

Crystal violet stain (1%): It composed of 0.5% (w/v) crystal violet and 50% methanol then made up to volume with ddH_2O and filtered through a Whatmann No.1 filter paper.

Cell line Propagation: The cells were propagated in Dulbecco's modified Eagle's medium (DMEM) supplemented with 10% heat-inactivated fetal bovine serum, 1% L-glutamine, HEPES buffer and 50µg/ml gentamycin. All cells were maintained at 37 ºC in a humidified atmosphere with 5% CO_2 and were sub-cultured two times a week.

Cytotoxicity evaluation using viability assay: For cytotoxicity assay, the cells were seeded in 96-well plate at a cell concentration of 1×10^4 cells per well in 100 µL of growth medium. Fresh medium containing different concentrations of the test sample was added after 24 h of seeding. Serial two-fold dilutions of the tested chemical compound were added to confluent cell monolayers dispensed into 96-well, flat-bottomed microtiter plates (Falcon, NJ, USA) using a multichannel pipette. The microtiter plates were incubated at 37 ºC in a humidified incubator with 5% CO_2 for a period of 48 h. Three wells were used for each concentration of the test sample. Control cells were incubated without test sample and with or without DMSO. The little percentage of DMSO present in the wells (maximal 0.1%) was found not to affect the experiment. After incubation of the cells for at 37 °C, various concentrations of sample were added, and the incubation was continued for 24 h and viable cells yield was determined by a colorimetric method.

In brief, after the end of the incubation period, media were aspirated and the crystal violet solution (1%) was added to each well for at least 30 minutes. The stain was removed and the plates were rinsed using tap water until all excess stain is removed. Glacial acetic acid (30%) was then added to all wells and mixed thoroughly, and then the absorbance of the plates were measured after gently shaken on Microplate reader (TECAN, Inc., Männedorf, Zürich, Switzerland), using a test wavelength of 490 nm. All results were corrected

for background absorbance detected in wells without added stain. Treated samples were compared with the cell control in the absence of the tested compounds. All experiments were carried out in triplicate. The cell cytotoxic effect of each tested compound was calculated**.** The optical density was measured with the microplate reader to determine the number of viable cells and the percentage of viability was calculated as [1-(ODt/ODc)]x100% where ODt is the mean optical density of wells treated with the tested sample and ODc is the mean optical density of untreated cells. The relation between surviving cells and drug concentration is plotted to get the survival curve of each tumor cell line after treatment with the specified compound. The 50% inhibitory concentration (IC_{50}) was estimated from graphic plots of the dose response curve for each conc. using Graphpad Prism software (San Diego, CA. USA) [44, 50].

44. Gomha, S. M.; Riyadh, S. M.; Mahmmoud, E. A.; Elaasser, M. M. *Chem. Heterocycl. Comp.*, **2015**, *51*, 1030–1038.

50. Gomha, S. M.; Riyadh, S. M.; Mahmmoud, E. A., Elaasser, M. M. *Heterocycles* **2015**, *91*, 1227.
